# “Implementation of intensified COVID-19 vaccination optimization (ICVOPT) campaigns strategy in complex humanitarian emergency settings and hard-to-reach areas; a case of South Sudan”

**DOI:** 10.1186/s12879-024-09371-4

**Published:** 2024-05-30

**Authors:** Dickens Atwongyeire, Ayesheshem Ademe Tegegne, Fabian Ndenzako, Brendan Dineen, Anson Edu Benjamin, Benz Luo, Ferede Melesachew, Emmanuel George Bachan, Andrew Baguma

**Affiliations:** World Health Organization, Central Equatoria State, Juba, South Sudan

**Keywords:** COVID 19 intensification, Optimization, ICVOPT, Complex humanitarian setting, South Sudan

## Abstract

**Introduction:**

The COVID-19 pandemic is still a public health concern in South Sudan having caused suffering since the first case of COVID-19 was introduced on 28^th^ February 2020. COVAX vaccines have since been introduced using a number of strategies including fixed site, temporary mobile, hit and run in flooded and conflict affected areas. We aim to describe the 2 ICVOPT campaigns that were conducted to improve the uptake and document lessons learnt during the initial rollout of the COVID-19 vaccination programin South Sudan between February 2022 and June 2022 each lasting for 7-days.

**Methodology:**

We conducted an operational cross-sectional descriptive epidemiological study of a series of the intensified COVID-19 vaccination Optimization (ICVOPT) campaigns from February 2022 to June 2022. Before the campaign, a bottom up micro-planning was conducted, validated by the County Health Departments (CHDs) and national MOH team. Each of the 2 campaigns lasted for 7 days targeting 30% of the eligible unvaccinated target population who were18 years and above. Each team consisted of 2 vaccinators, 2 recorders and 1 mobilizer. The teams employed both fixed site, temporary mobile, hit and run in flooded and conflict affected areas. The number of teams were calculated based on the daily workload per day (80 persons per team/day) for the duration of the campaigns.

**Results:**

A total of 444,030 individuals were vaccinated with primary series COVID-19 vaccine (J&J) out of the targeted 635,030 persons. This represented 69.9% of target population in the selected 28 counties and 10 states of South Sudan in 7 days’ ICVOPT campaigns. More eligible persons were reached in 7 days campaigns than the 9 months of rollout of the COVID-19 vaccine prior to ICVOPT campaigns using the fixed site strategy at the health facility posts.

**Conclusion:**

Intensified COVID-19 vaccination Optimization (ICVOPT) campaigns were vital and fast in scaling up vaccination coverages as compared to the fixed site vaccination strategies (2022 progress report on the Global Action Plan for Healthy Lives and Well-being for All Stronger collaboration for an equitable and resilient recovery towards the health-related Sustainable Development Goals, incentivizing collaboration, 2022) in complex humanitarian emergency settings and hard-to-reach areas of South Sudan.

## Introduction

“Coronavirus disease (COVID-19) is an infectious disease caused by the SARS-CoV-2 virus. The virus was first reported from Wuhan city in China in December, 2019, which in less than three months had spread throughout the globe. It was declared a global pandemic by the World Health Organization (WHO) on 11th of March 2020 [1].

South Sudan was equally affected by the pandemic with the Ministry of Health declaring the first 29-year old female, a returnee COVID-19 patient from the Netherlands on 28 February 2020 via Addis Ababa. She presented with fever, cough, headache and shortness of breath, subsequently laboratory confirmed on the 4th April 2020. Several activities were undertaken including screening using infra-red thermometers, quarantine and isolation of returnees from the highest risk countries at the time, enforcing total lockdown. In addition, curfews, travel restrictions, simple day-to-day measures like regular hand washing with soap and water; sanitizers, practicing cough hygiene, maintaining a social distance of at least one meter [1] were also utilized. In Africa, the first deliveries of the COVAX facility vaccines were registered in Ivory Coast and Ghana by February 24th 2021 which marked the start of what would be the largest, most rapid and complex global rollout of COVID-19 vaccines in history [2]. Thereafter, through the COVAX facility, vaccines were secured and deployed on 7th April 2021 in South Sudan, employing a number of strategies in the rollout of COVID-19 vaccination programme. The country employed fixed site strategy where vaccination activities were localized at the health facility. COVID-19 vaccination had been used like the rest of the world to bring the effects of the COVID-19 pandemic under control, in line with WHO’s 5 core components of COVID-19 preparedness, readiness and response [3]. The need to scale up COVID-19 vaccine uptake in South Sudan became necessary and of concern to WHO AFRO as it was evident that from April 2021 to December 2021 the cumulative percentage vaccination coverage was less than 2% and therefore failed to hit its WHO global target of 40% by the end of December 2021 [4]. That situation placed the country amongst the last three poorly performing countries in the continent of Africa [5]. This had been blamed on low risk perception by the population, lack of trust in government programs, misconceptions against COVID-19 vaccines causing infertility [6]. The vaccine was also associated with “sign of the beast” mentioned in the bible and number 666, infodemics from social media platforms such as recipients dying after 2 years post vaccination among others [6]. To make considerable progress, The World Health Organization Regional Office for Africa (WHO AFRO) supported the member states Ministries of Health with financial and human resources in form of technical assistance in driving the COVID-19 response in Africa. This gave South Sudan in particular an opportunity to employ a campaign mode of service delivery, Intensified COVID-19 vaccination Optimization (ICVOPT) with support from WHO South Sudan. The strategy employed multi-stakeholder approach to ensure political, social, community participation which enhanced programme ownership and possible future sustainable programming by integrating it into existing EPI structures and PHC services [3]. The strategy was able to bring vaccination services close to the most affected population by using mobile outreaches and hit and run modalities- employed in the security compromised states of Unity, Lakes, Upper Nile and Jonglei; due to civil conflict, cattle rustling and “revenge killing” and severe episodes of flooding. A group of vaccination teams would mobilize communities through their leaders to enable them access life saving services in a short period of time and then they would retreat afterwards to safer areas.

It is important to note that these states and counties affected by armed conflict, flooding and severe food shortages suffered from poor COVID-19 vaccination coverage rates.This is consistent with the UN security council report that vaccination rates in conflict-affected countries and areas facing humanitarian crises tended to be particularly low, as they faced additional challenges in planning, delivering critical services and administering the vaccines to the most vulnerable populations that need the jabs [7]. The report further goes on to highlight that as of 24th March 2022, Burundi and Haiti had received enough doses to cover only 3% of their populations; the Democratic Republic of the Congo and Yemen, 4%; Cameroon, 6% whereas Mali and South Sudan (8%) eight% [7]. The report emphasized that Syria had received enough doses to vaccinate 35% of its population and Nigeria 17%, slightly better than their humanitarian context affected counterparts. Much as planning, delivery and administering the vaccines seemed to be severely affected equally in these countries, some countries are more affected than others [7]. The factors responsible for low coverages included limited logistical capacity, humanitarian access challenges, fragmented healthcare systems, low capacity of government to train, recruit and sustain qualified health care workers that are well remunerated, which causes chronic brain drain and consequently shortage of healthcare workers [7, 8]. Complicated issues related to poor access to eligible vulnerable populations remained a challenge for such countries such as Syria. Even though countries affected by humanitarian crises are fully supported by the concerted COVID-19 vaccination delivery partnership (CoVDP) and COVAX facility partners, absorption of these vaccines remains low. Issues around hesitancy remain due to difficulties in reaching appropriate information Education and communication (IEC) materials and access to the community leaders and communities in its entirety. Observations have been made that trust in fellow community mobilization members, community members and political leaders from such communities appeared to be low which further exacerbated the low coverage rates [7]. This affected the quality of COVID-19 vaccination activities offered and also the quality of data captured and eventually reported into the reporting databases. Due to protracted conflicts, there is usually weak governance, mistrust of government programs and eventually mistrust and or distrust of information like this one on COVID-19 and its vaccines [6]. The lower rates of vaccination may be also explained by skeptical attitudes towards vaccination making them susceptible to vaccine hesitation which can be addressed with clear, accessible and tailored information campaigns way beyond the COVID-19 vaccination campaigns period so that all the myths, misinformation and rumors can be counteracted by well-structured and informed community leadership structures [9, 10].

The purpose of this paper is to describe the ICVOPT campaigns that were conducted to improve uptake and provide evidence based information that could be used in similar settings to rollout COVID-19 vaccination and other influenza like illnesses vaccinations. We aim to describe the 2 ICVOPT campaigns that have been conducted to improve the uptake and document lessons learnt during the initial rollout of the COVID-19 vaccination program in South Sudan between February 2022 and June 2022 during the 7-days campaigns.

## Methodology

### Study setting

South Sudan is a landlocked country located in East Africa covering approximately 640,000km^2^ with a projected population of 13,154,416 and a population density of 18 per square kilometer. The country is divided into ten states, 3 administrative units, 80 counties and 507 Payams. The country became independent on 9th July 2011, however, the country has struggled with tribal clashes and armed conflict, undeveloped road infrastructure that is even made worth by rains, causing massive floods, population movements and disruptions during the rainy seasons to the detriment of the health services and health indicators in the country.

### Study design

We conducted a retrospective descriptive analysis of ICVOPT campaigns conducted in South Sudan from February 2022 and June 2022 amongst adult persons aged 18 years and above according to the South Sudan Immunization Technical Advisory Group (SSITAG) recommendations on COVID-19 Vaccine policy in the country.

### Study description

An operational Cross-sectional descriptive epidemiological analysis of a series of the Intensified COVID-19 Vaccination Optimization (ICVOPT) campaigns was conducted in February 2022 and June 2022 during the 7 days campaigns. A total of 28 counties of the 80 counties were selected based on availability of funds, hard to reach status and low vaccination coverages at the time.

The coordination and planning meetings were held in the selected States and counties based on high number of unvaccinated persons, hard to reach counties as follows: 1st phase of ICVOPT; Northern Bahr El Ghazal (Aweil Centre), Unity (Rubkhona), Eastern Equatoria (Torit, Magwi), Western Bahr El Ghazal (Jur River, Wau) Central Equatoria (Juba). 2nd phase ICVOPT; Upper Nile (Manyo, Fashoda, Maban, Baliet, Akoka), Jonglei (Bor South, Twic East, Duk, Pibor), Lakes (Cueibet, Rumbek East, Awerial, Yirol west), Unity (Guit, Pariang, Mayom, Panyijar), Warrap ( Twic, Gogrial West, Gogrial East). Bottom up planning was facilitated by a robust WHO EPI and state coordination mechanisms (WHO EPI officers, EPI field national supervisors and Health Education and Promotion (HEP) officers were selected by Ministry of Health (MOH), oriented and deployed to support different areas of technical and risk communication and community engagement at state and county levels. Each county was supported by one EPI national supervisor and one HEP officer to support the county health department (CHD) in risk communication and community engagement activities. These campaigns were resourced through WHO direct implementation mechanisms. WHO States Coordination offices were urged to support all preparations. Activities supported included microplanning support, training of the vaccinators, recorders, social mobilizers, community meetings and other advocacy meetings like launch ceremonies for governance and leadership buy-ins, building trust by communities in the vaccine towards uptake of the COVID-19 vaccine. The vaccination sessions consisted of 5 team members: 2 vaccinators, 2 recorders and 1 social mobilizer. The vaccination activities took an average of 7 days at designated vaccination posts but the vaccination teams were allowed to change locations (mobile and temporary vaccination posts) to new areas whenever the primary locations were exhausted of the people in need of the vaccinations. The team supervisor in liaison with the social mobilizer mobilized the communities where they were going next through their community leaders for vaccination. Vaccination teams took advantage of the different humanitarian activities like Insecticide treated nets (ITNs) distribution, food distribution, health camps, cattle camps, market areas, churches and waterpoints to position the vaccination posts. The strategy also used Focus group Discussion (FGD) meetings with women groups, youth leaders, youth groups, religious leaders and community leaders in all the counties reached.

### Data sources

All the data compiled was derived from the South Sudan COVID-19 vaccination dashboard at national level and reports provided by the COVID-19 vaccination team. A compilation of data occurred at the vaccination site level using tally sheets, daily summaries and sent it to the team supervisor who oversaw 3 vaccination teams, who in turn summarized daily teams’ summaries, reported it to the data manager at the County Health department (CHD) to be entered into the Open Data Kit (ODK) online tool. This data would then be received at the national level, verified and cleaned accordingly.

### Analysis

**Fig. 1:**
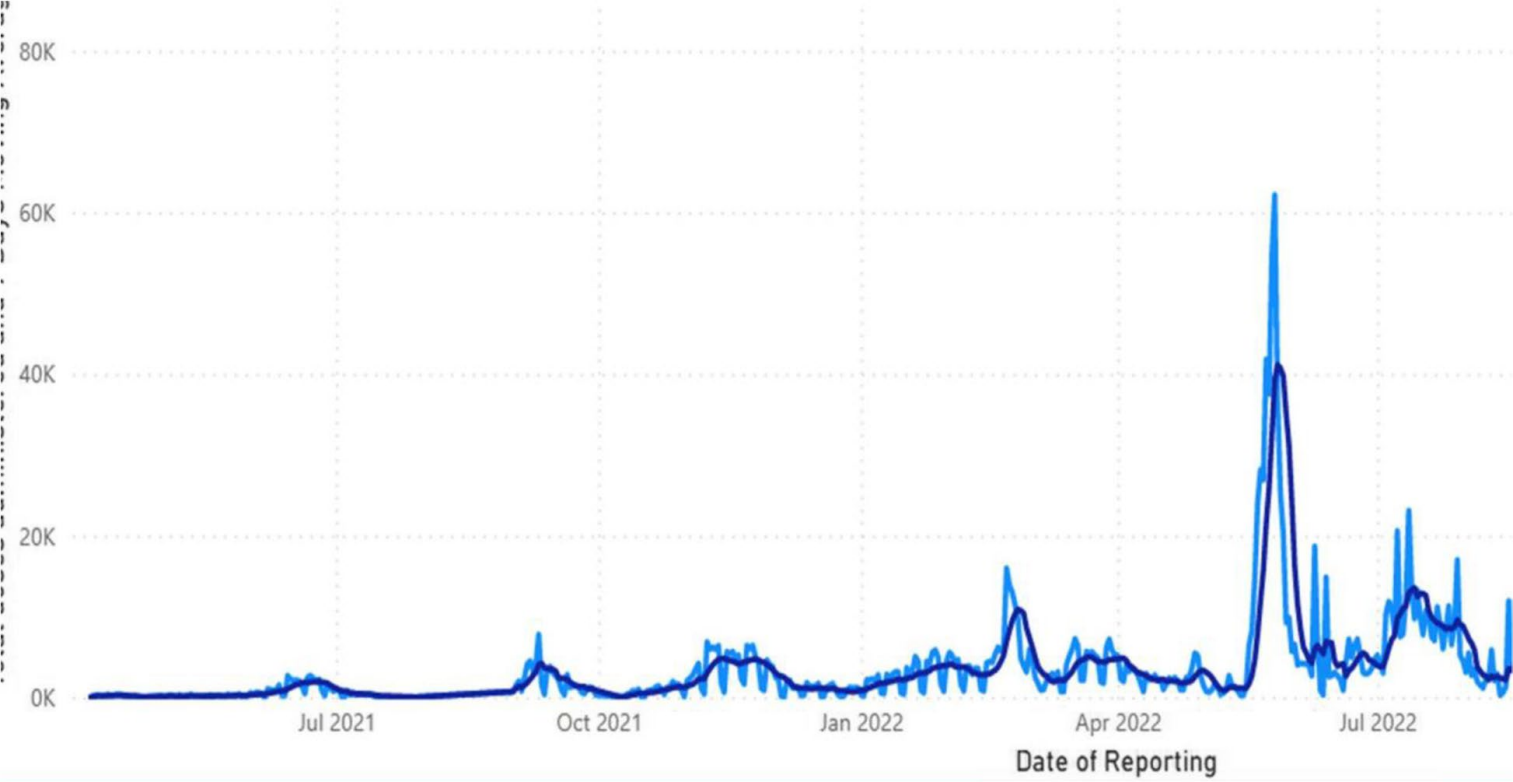
A graph showing the contribution of the 2 phases of ICVOPT campaigns towards scale up of COVID-19 Vaccination between Feb-June 2022, South Sudan

**Fig. 2:**
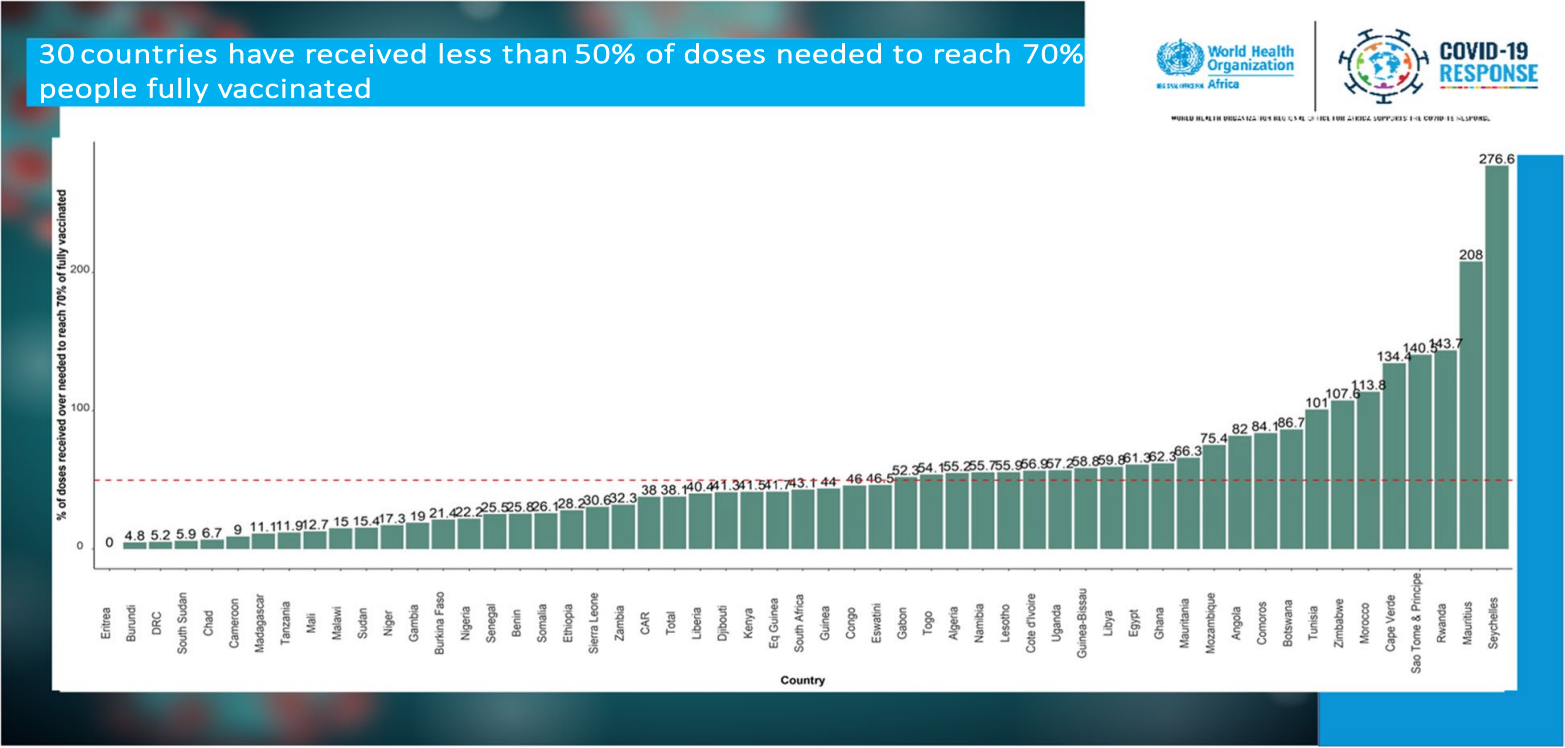
A graph showing South Sudan’s low performance during early rollout of vaccination programme in 2022

We conducted a basic analysis from the available information using excel spreadsheet to conduct descriptive analysis and produce graphs (Figs. 1 and 2) and tables (Tables 1, 2 and 3) from the South Sudan COVID-19 vaccination dashboard and reports from the COVID-19 vaccination team of South Sudan.

**Table 1:** Table showing performance of 5 States and 7 counties during the ICVOPT campaign in February 2022, South Sudan

State	County	Target Population	# Population fully vaccinated	% Vaccinated persons
Northern Bahr El Ghazal	Aweil Centre	12,941	11,713	90.5
Unity	Rubkhona	19,268	18,617	96.6
Eastern Equatoria	Torit	17,657	11,545	65.4
	Magwi	28,107	6,978	24.8
Western Bahr El Ghazal	Jur River	20,000	9,931	49.7
	Wau	8,014	6,229	77.7
Central Equatoria	Juba	15,283	4,540	29.7
**Total**		**121,268**	**69,553**	**62.0**

**Table 2:** Table showing performance of 5 States and 21 counties during the ICVOPT campaign in June 2022, South Sudan

State	Counties	Target Population	# Population vaccinated	% COVID-19 vaccinated
Upper Nile	Maban	32,696	24,404	74.6
	Fashoda	2,229	5,314	238.4
	Manyo	5,734	4,668	81.4
	Akoka	4,396	4,358	99.1
	Baliet	3,775	3,834	101.6
	**Total**	**48,830**	**42,578**	**87.2**
Warrap	Gogrial west	55,087	53,916	97.9
	Gogrial East	22,157	22,048	99.5
	Tonj North	43,896	32,031	73.0
	Twic	46,944	44,900	95.6
	**Total**	**168,084**	**152,895**	**91.0**
Lakes	Awerial	18,084	4,922	27.2
	Rumbek East	32,106	22,260	69.3
	Cueibet	27,288	22,775	83.5
	Yirol West	24,565	18,667	76.0
	**Total**	**102,043**	**68,624**	**67.3**
Jonglei	Bor South	30,382	6,812	22.4
	Duk	9,966	2,474	24.8
	Pibor	52,987	33,975	64.1
	Twic East	7,139	905	12.7
	**Total**	**100,474**	**44,166**	**44.0**
Unity	Guit	14,189	12,395	87.4
	Mayom	29,776	26,082	87.6
	Panyijar	12,575	7,847	62.4
	Parieng	37,791	19,890	52.6
	**Total**	**94,331**	**66,214**	**70.2**
	**GRAND TOTAL**	**513,762**	**374,477**	**72.9**

**Table 3:** Table showing the performance of the respective counties before and after the ICVOPT campaigns in South Sudan, 2022

SNO	County	Absolute # vax. before ICVOPT	% Before ICVOPT	Absolute # vax. after ICVOPT	% after ICVOPT	% rise after ICVOPT
1	Aweil Centre	3,671	11.99	17,792	58.1	46.11
2	Rubkhona	6,607	8.31	41,979	52.82	44.51
3	Torit	2,742	3.36	20,378	24.96	46.11
4	Magwi	2,801	2.02	15,696	11.31	9.29
5	Jur River	4,527	4.82	23,897	25.44	20.62
6	Wau	17,562	15.77	30,855	27.71	11.94
7	Juba	48,839	17.94	75,415	27.71	9.77
8	Maban	525	1.54	31,991	94	92.46
9	Fashoda	1,217	4.42	9,043	32.86	28.44
10	Manyo	238	0.84	8,058	28.53	27.69
11	Akoka	219	3.03	5,001	69.11	66.08
12	Baliet	-	0	4,056	14	14
13	Gogrial West	3,025	1.69	60,219	33.63	31.94
14	Gogrial East	2,822	3.73	25,430	33.63	29.9
15	Tonj North	3,323	2.74	38,248	31.54	28.8
16	Twic	4,753	3.16	50,475	33.53	30.37
17	Awerial	658	1.72	7,137	18.71	16.99
18	Rumbek East	899	0.91	25,705	25.88	24.97
19	Cueibet	3,095	3.26	26,790	28.25	24.99
20	Yirol West	1,975	2.38	22,569	27.18	24.8
21	Bor South	2,351	1.5	9,813	6.28	4.78
22	Duk	204	0.44	2,839	6.17	5.73
23	Pibor	1,098	1.05	35,818	34.27	33.22
24	Twic East	410	0.68	1,462	2.42	1.74
25	Guit	1,230	8.31	14,279	54.86	46.55
26	Mayom	1,243	1.3	28,888	30.17	28.87
27	Panyijar	733	1.81	9,608	23.77	21.96
28	Pariang	5,122	7.82	32,748	49.97	42.15
	**Total**	**121,889**	**4.16**	**676,189**	**32.39**	**29.1**

## Results

In February 2022, 69,553 persons were vaccinated with primary series COVID-19 vaccine (J&J), whereas a total of 374,477 persons were vaccinated after the second ICVOPT campaign in June 2022, making a total of 444,030 individuals who had been reached with COVID-19 vaccine (J&J). This was against the 635,030 persons targeted between the February 2022 and June 2022, representing 69.9% of target population in the selected 28 counties and 10 states, which raised the coverage to 29.1% (Tables 1 and 2). It is worth noting that some counties though improved in coverages after the intensified campaigns, some of them were still below the required standards. These were Magwi (25%), Twic East (13%), Jur River (50%), Juba (30%), Duk (25%), Bor South (22%), Awerial (27%), Parieng (53%). It was observed that low risk perceptions, hesitancy among the youth populations through social media, poor coordination from the vaccine hesitant county and states administrators contributed immensely to the low performance. Consideration of the vaccinated persons before and after the ICVOPT campaigns indicated that averagely 29% more persons were vaccinated with the COVID-19 vaccine. Some counties registered better performance than their counterparts as shown in Tables 1 and 2.

### Data as of 27th June 2022 from South Sudan COVID-19 vaccination dashboard

This graph highlights the chronology of events from when vaccination rollout began in April 2021 up to when the Ministry of Health (MOH) in collaboration with WHO and partners initiated the 2 phases of Intensified COVID-19 Vaccination Optimization campaigns to help scale up vaccination coverages between February 2022 and June 2022. This is indicated by sharp spikes in performance.

This graph illustrates the performance of South Sudan in COVID-19 vaccination as compared to other African states during early 2022. South Sudan was one of the lowest performing countries in the rollout of the COVID-19 vaccination services prior to this ICVOPT initiation.

## Discussion

In our study, we found out that some counties had performed above 60% of the target population (Akoka and Maban), while Magwi (25%), Twic East (13%), Jur River (50%), Juba (30%), Duk (25%), Bor South (22%), Awerial (27%), Parieng (53%) performed < 60% of the micro plan target. Good performance in some counties could be attributed to better leadership advocacy, social mobilization and community engagement at all levels, presence of IDPs and refugees in respective counties and presence of enabling health infrastructure and supportive cluster partners. Poor performance in some counties could be associated with some of the highlighted factors. There were numerous instances of insecurity (shooting, vaccinator arrests leading to confiscation of the vaccines and data tools) that led to threats, intimidation, neglecting of vaccination sites, harassments to the communities and the vaccination teams that were observed in many areas leading to lower COVID-19 vaccination coverages in those Payams and counties. The lower the vaccination coverages the lower the community herd immunity attained to protect them from the negative impacts of COVID-19 pandemic like severe illness, hospitalizations and deaths.

It is important to note that various areas had not been reached optimally due to the inaccessibility challenges because of inter-tribal clashes [11–13]. This was related to other humanitarian challenges like flooding, severe drought associated with severe food insecurities that had devastated some parts of the country putting the COVID-19 vaccination never number one priority. Poor penetration of information into the communities had been attributed to shortage of risk communication and community engagement rendering them hesitant to the vaccine, partly due to shortage of megaphone and batteries where some of them would do social mobilization activities without megaphones. High levels of hesitancy in some communities had been attributed to low risk perception levels to the COVID-19 pandemic itself and hence poor uptake of the vaccines. Some areas however lacked optimal coordination mechanisms, multiple infodemics circulating on series of social media outlets persisted in most counties and Payams [14]. These infodemics have been associated with saturation of some communities with negative information on COVID-19 vaccine that led to deep rooted anti-COVID-19 vaccination propaganda by some overzealous religious and traditional leaders especially in Jonglei state. These included “Infertility in the reproductively active young persons, “vaccine recipients must die within two years of the receiving the vaccine”, the vaccines cause blood clots, vaccine was connected with the biblical concept of “illuminati” from a misconception related to toll free number ‘6666’ and biblical ‘666’ in the Holy Bible citing Revelations 13: 16–17 verses as confirmation [14, 15].

We aim to describe the 2 ICVOPT campaigns that have been conducted to improve the uptake and document lessons learnt during the initial rollout of the COVID-19 vaccination program in South Sudan between February 2022 and June 2022 in each of the 7-days campaigns.

### Lessons learned and good practices

The bottom-up microplan development & engagement of counties’ leaderships during planning and implementation motivated teams at respective levels to work harder to perform better than was expected [12, 16]. Strong leadership support, commitment & engagement during pre, intra & post campaign activities with States, County, Payam, Bomas provided a platform for reaching out to the communities with COVID-19 vaccination services as witnessed during ICVOPT [17]. This helped in reaching out to the vulnerable populations like elderly persons, IDPs/Refugees, females by bringing COVID-19 vaccination services ‘’closer to the people” and communities alike [17] Early initiation of Social mobilization activities including engagement of the churches, mosque and community leaders, youth and women groups at Payams and Bomas improved awareness about COVID-19 vaccination. The RCCE teams also engaged youths, women groups through Focused Group Discussions (FGDs), community dialogue meetings and held one on one advocacy meetings with their leaders to cascade awareness to their community members [17]. Regular daily coordination meetings (Performance Review meetings) at the different coordination levels like States, Counties, Payams aimed at daily review of the teams performance, identified gaps and devised appropriate corrective actions for improved performance during prospective vaccination campaigns, playing a key role in achieving the objectives of the ICVOPT campaigns. Vaccination teams took advantage of the different humanitarian activities like Insecticide treated nets (ITNs) distribution, food distribution, health camps and other community congregational points like market areas, churches and water points to position the vaccination posts. The strategy also used FGD meetings with women groups, youth leaders, youth groups, religious leaders and community leaders in all the counties reached. These interventions if utilized well in complex humanitarian emergency settings and hard-to-reach areas in collaboration with community leaders lead to improved response to crises and disasters in similar humanitarian settings.

## Conclusion

Countries operating in humanitarian settings face a multitude of challenges in rolling out vaccination programmes. Intensified COVID-19 vaccination Optimization (ICVOPT) campaigns were vital and fast in scaling up vaccination coverages as compared to the fixed site vaccination strategies [18] in complex humanitarian emergency settings and hard-to-reach areas of South Sudan. Several ICVOPT campaigns were necessary to raise the COVID-19 vaccination coverages to build a sustainable community herd immunity against the COVID-19 pandemic in the country.

### Limitations of the study

The study analyzed challenges, good practices and lessons learned during implementation of vaccination activities for use by members states experiencing similar humanitarian settings. By virtue of the complex fragile humanitarian context and weakened health systems some information may not be available, hence making it difficult to conclude on the effects of this innovation.

## Data Availability

Data is publicly available for information. The datasets generated and/or analyzed during the current study are available in the “Republic of South Sudan Ministry of Health COVID-19 Vaccination dashboard and reports” repository.

## Abbreviations

CHDs: County Health Department
COVDP: COVID-19 Vaccine Delivery Partnership
COVID-19: Corona Virus Disease 2019
EPI: Expanded Programme on Immunization
FGD: Focus Group Discussions
ICVOPT: Intensified COVID-19 vaccination Optimization
IEC: Information Education and Communication
ITNs: Insecticide Treated Mosquito Nets
MOH: Ministry of Health
RCCE: Risk Communication and Community Engagement
UN: United Nations
AFRO: Regional Office for Africa
WHO: World Health Organization
